# HRK inhibits colorectal cancer cells proliferation by suppressing the PI3K/AKT/mTOR pathway

**DOI:** 10.3389/fonc.2022.1053510

**Published:** 2022-12-07

**Authors:** Haowei Wang, Yujia Chen, Qinzi Yuan, Lixia Chen, Peiling Dai, Xuenong Li

**Affiliations:** ^1^ Guangdong Provincial Key Laboratory of Molecular Tumor Pathology, Department of Pathology, School of Basic Medical Sciences, Southern Medical University, Guangzhou, China; ^2^ Department of Pathology, Zhujiang Hospital, Southern Medical University, Guangzhou, China; ^3^ Department of Pathology, Nanfang Hospital, Southern Medical University, Guangzhou, China

**Keywords:** HRK, CRC, mTOR, rapamycin, proliferation, apoptosis

## Abstract

**Background:**

As one of the most common malignant tumor, colorectal cancer (CRC) continues to have a high incidence and mortality rate. HRK belongs to the BCL-2 protein family, which has been shown to have antitumor effects in prostate cancer. However, its role in colorectal cancer is not yet known.

**Methods:**

In this study, we verified the expression levels of HRK in colorectal cancer tissues by public database search as well as immunohistochemistry. Next, we analyzed HRK expression levels in CRC tissues,adjacent non-cancerous tissues, cell lines and normal intestinal epithelial cells by qPCR and Western blotting. CCK-8 proliferation assays, transwell assays, wound healing assays, colony assays and flow cytometry were performed to clarified the effect of HRK on CRC cells. Western blotting and rescue experiments were used to determine the role of HRK in regulating PI3K/AKT/mTOR signaling pathway.

**Results:**

HRK expression was lower in CRC tissues and cell lines. Gain and loss of function experiments showed that HRK decreased proliferation, invasion and migration of CRC cells. Low expression of HRK inhibited CRC cell apoptosis as well as activated the PI3K/AKT/mTOR signaling pathway. In addition, rapamycin inhibits the activation of PI3K/AKT/mTOR signaling pathway and reverses HRK-induced alterations in cell biological functions.

**Conclusion:**

Our study demonstrates that HRK is lowly expressed in colorectal cancer tissues. And for the first time, HRK was shown to promote apoptosis and inhibit proliferation of colorectal cancer cells by inhibiting PI3K/AKT/mTOR signaling pathway. HRK represents a potential target for the treatment of CRC.

## Introduction

Colorectal cancer (CRC) is the third most commonly diagnosed cancer and the second most common cause of cancer death worldwide (1). The initial events in CRC tumorigenesis have been relatively well-studied, and treatments for early-stage disease have significantly improved over the past decades, however, the metastatic disease accounts for 40% to 50% of newly diagnosed patients, which is associated with high morbidity (2). Despite the continuous improvement of treatment methods and tools, the 5-year survival rate of patients with advanced stages has been hovering around 10%. Most patients with early-stage colorectal cancer are cured by surgery to remove the primary lesion. However, surgical outcomes are not satisfactory for patients with intermediate to advanced stages, leaving approximately 30% of stage II-III patients and 65% of stage IV patients at the risk of recurrence. Chemotherapy is an important treatment for advanced and metastatic CRC, but the drug resistance of tumor cells leads to poor outcomes in most patients. In recent years, some progress has been made with targeted therapies and immunotherapy, but less than one-third of patients benefit from these treatments. Currently, most patients with advanced and metastatic CRC still lack effective therapeutic agents (3–5).

Akt is the central mediator of the PI3K pathway. Phosphorylated-Akt activates downstream effector mTOR through TSC1/2 (tuberous sclerosis 1/2 complex) and upregulates various transcription factors to promote protein synthesis, cell growth, cell survival and motility. On the other hand, activated mTORC2 (mTOR complex 2) phosphorylates Akt at ser473, which also leads to hyperactivation of Akt (6, 7). Several studies have shown that the PI3K/Akt/mTOR pathway is frequently genetically altered in human cancers.

Harakiri (HRK), located on chromosome 12, belongs to a subgroup of the Bcl-2 family proteins containing only the BH3 structural domain. It is clear from yeast two-hybrid experiments that HRK binds to Bcl-2 and Bcl-xL (8–11). Apoptosis is activated by Exogenous expressed HRK, an effect that can be inhibited by overexpression of Bcl-2 and Bcl-xL (12). It has also been documented that Bax is a direct effector molecule of HRK action (13). However, the exact molecular mechanisms implicated in HRK-mediated apoptosis remain unknown.

Early studies found that low expression of HRK promotes the development and progression of cancer (14). In colorectal cancer, gastric cancer and prostate cancer, HRK is often at low or absent expression level (15–17). Yes-associated protein (YAP) causes tumor invasion and chemoresistance through inhibition of HRK expression in neuroblastoma (18). On the other hand, HRK in glioblastoma, non-small lung cancer, breast cancer as well as ovarian cancer inhibits tumor proliferation by promoting apoptosis (11, 19–22). Despite its potential significance, the role of HRK function has not been well-defined in cancer.

In this study, we aimed to investigate the expression level of HRK in colorectal cancer and to demonstrate that the effect of HRK on the biological function of colorectal cancer cells is regulated through the PI3K/AKT/mTOR pathway. We hope that our study will help to identify potential therapeutic targets for the treatment of CRC or to improve the diagnosis of colorectal cancer.

## Materials and methods

### Tissue specimens and cell lines

Colorectal tumor tissue samples were obtained from patients who had been pathologically diagnosed with primary colorectal carcinoma in Nanfang Hospital, Southern Medical University. No patient received any chemotherapy and radiotherapy before operation. Freshly frozen samples from CRC patients were used for real-time PCR and western blotting. Formalin-fixed tumor tissues specimens, comprising 31 CRC tumor tissues and 41 adjacent non-tumors tissues, were used for IHC analysis.

The immortalized colon mucosa epithelial cell line (FHC, CRL-1831), human colorectal carcinoma cell lines RKO (CCL-2577), DLD1 (CCL-221), HCT116 (CCL-247), SW480 (CCL-228), SW620 (CCL-227), LoVo (CCL-229), HCT8 (CCL-244), and HCT15 (CCL-225) were obtained from the American Type Culture Collection (ATCC).

All CRC cell lines were cultured in RPMI 1640 medium (Gibco, Gaithersburg, MD, USA) with 10% fetal bovine serum (FBS, Gibco). FHC cells were cultured in DMEM: F12 medium (Gibco) with 10% FBS. All of the cell lines were cultured at 37°C and in 5% CO_2_ in air in a humidified incubator. Rapamycin (TOPSCIENCE, China) applied at a concentration of 100 nM for 24 h was used as an mTOR inhibitor.

### RNA isolation and real-time RT-PCR

Total RNA were isolated based on the manufacturer’s protocol of TRIzol Reagent (TaKaRa, Dalian, China). cDNA was converted by PrimeScript RT Reagent Kit (TOYOBO). Real-time RT-PCR was carried out on an ABI PRISM 7500 Sequence Detection System (Applied Biosystems, Foster City, CA) using Bestar^®^ SybrGreen qPCR mastermix (DBI). The reference gene was GAPDH. The assay was performed in triplicate for each case to allow the assessment of technical variability. Comparative quantification was determined using the 2-ΔΔCt method. All primer sequences are listed in Supporting Information (Data S1).

### Western blotting analysis

Prechilled RIPA buffer containing phosphatase inhibitors and protease inhibitors was used to lyse Cells and tissues. The protein lysates were separated by SDS-PAGE and transferred to PVDF membranes (Millipore, Darmstadt, Germany). The membranes were blocked in 5% skimmed milk in 1x TBS-T (0.5% Tween-20) and incubated overnight at 4°C with the following primary antibodies: HRK (1:500; immunoway), CCND1, CDK4, CDK6, p21, BAX, Bcl-2, cleaved caspase-3, caspase-3, AKT, p-AKT(Ser473), p-PI3K(Tyr458), PARP1, cleaved-PARP1, GAPDH (1:1000; Proteintech), mTOR, p-mTOR(Ser2448), PI3K (1:1000; ZEN BIO). P70S6K, p-P70S6K(1-T389) (1:1000; Abclonal). After incubated with HRP-conjugated secondary antibodies for 1 h at room temperature, the membranes were visualized with ECL Western Blotting Substrate (FDbio). Immunoblotting signals were detected by densitometry using Image J software.

### Immunohistochemistry analysis

Immunohistochemistry (IHC) was performed with the following modifications. The slides were incubated overnight with primary antibody HRK (1:500, Abcam) at 4°C. Immunodetection was performed using diaminobenzidine (DAB) (ZSGB-BIO) according to the manufacture’s protocol. The IHC score were evaluated by two pathologists. Scores were determined based on both the intensity and percentage of HRK-positive cells. The intensity of IHC staining was designated as: 0 (no staining), 1 (weak staining), 2 (moderate staining), and 3 (strong staining). The percentage of stained cells was determined as: 1 (1–25%), 2 (26–50%), 3 (51–75%), and 4 (76–100%). The final score was defined as the staining number score multiplied by the staining color score. A score of <6 refers to the low expression group and ≥6 to the high expression group.

### Construction of cell lines with overexpressed and downregulated HRK

The full-length human HRK with 360-bp DNA fragment amplified and cloned into pcDNA3.1 (+) vector (GENEWIZ). The plasmid was transfected with the lipofectamine 3000 reagent (Invitrogen ThermoFisher Scientific). The empty vector pcDNA3.1(+) was used as control. Three siRNA sequences specifically targeting HRK and a control siRNA (Data S2) were designed and synthesized (RiboBio). The most effective siRNA sequence were infected with the lipofectamine 3000 reagent (Invitrogen ThermoFisher Scientific) in achieving knockdown of HRK was selected by Real-time RT-PCR and Western blotting.

### *In vitro* assays for evaluation of cell survival, proliferation, migration and invasion

Cell proliferation, colony formation, wound-healing, invasion assays, cell apoptosis, and flow cytometry cell cycle were performed *in vitro* according to standard protocols. Details are described in Supporting Information (Data S3). All experiments were performed in triplicate.

### Statistical analysis

All statistical analyses were performed using the GraphPad Prism 7 (GraphPad, USA). Student’s t-test (two tailed) or one-way ANOVA were used to calculate the difference between two groups or more than two groups. Pearson’s χ^2^ test was used to calculate the correlation between HRK mRNA expression levels and clinicopathologic features of CRC patients. Survival curves was estimated by Kaplan–Meier method (the log-rank test). The multivariate survival analysis was performed using Cox regression model. A probability value of 0.05 or less was considered as significance.

## Results

### HRK is downregulated in CRC and associated with tumor progression and prognosis in patients

**Figure 1:**
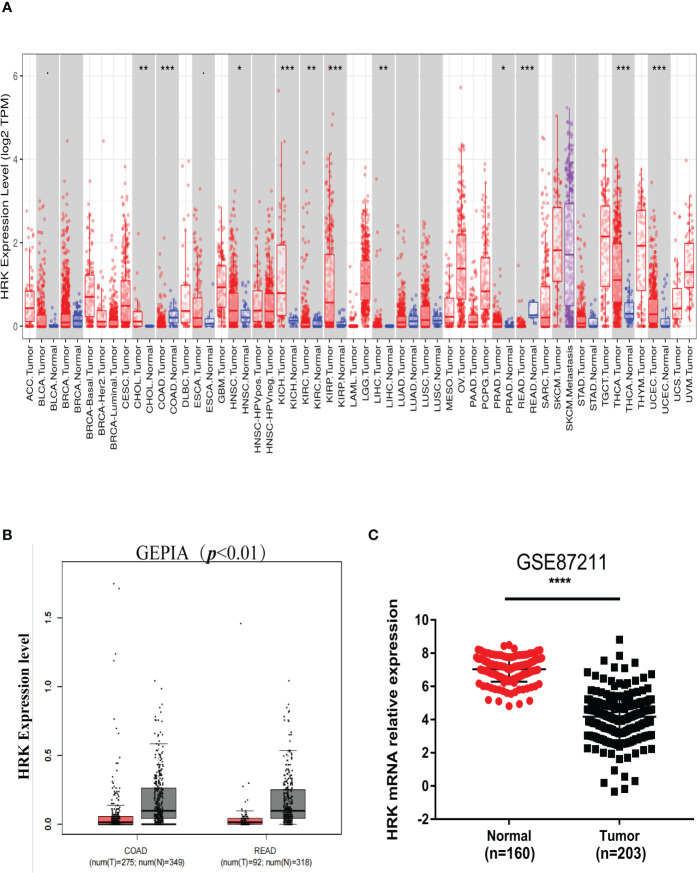
HRK expression levels in cancers. **(A)**, Human HRK expression levels in different types of tumors from TCGA data in TIMER (TCGA, the cancer genome atlas; TIMER, tumor immune estimation resource). **(B)**, Human HRK expression levels in Rectal adenocarcinoma (READ) and Colon adenocarcinoma (COAD) TCGA data in GEPIA (red bar: tumor, grey bar: normal, P < 0.01). **(C)**, Expression of HRK mRNA in cancerous and normal tissues in the GSE87211 database. The error bars represent mean ± SD. *P <0.05, **P <0.01, ***P <0.001 and ****P <0.0001.

**Figure 2:**
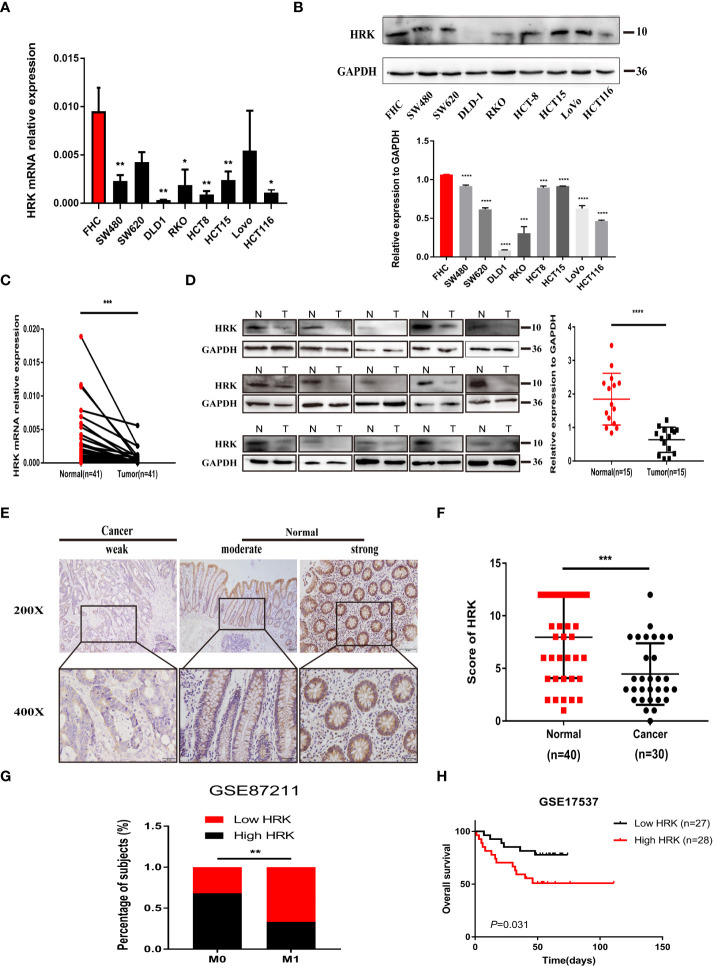
HRK is downregulated in CRC and likely predicted good prognosis in CRC patients. **(A, B)**, Expression levels of HRK mRNA **(A)** and protein **(B)** in CRC and colon mucosa epithelial cell lines. **(C, D)**, Expression levels of HRK mRNA **(C)** and protein **(D)** in paired CRC and adjacent noncancerous tissues (N: Normal, T: Tumor). **(E)**, Expression analysis of HRK protein in CRC and normal colorectal mucosa tissues by IHC. Scale bars, 50 μm (×200 magnification) or 20 μm (×400 magnification). **(F)**, Distribution of HRK expression in cancerous and normal tissues in IHC. **(G)**, Percentage of high and low expression of HRK with different distal metastasis in the database GSE87211 (M0: the patient has no distant metastasis, M1: metastasis to one or more distant sites or organs, or peritoneal metastasis is confirmed). **(H)**, Kaplan–Meier survival analysis of in database GSE17537 with low and high expression of HRK. Data information: Graphs report mean ± SEM. Significance was assessed using two-tailed Student’s t test, except for G where Chi-square test was used and H where log-rank test was used. *P < 0.05, **P <0.01, ***P < 0.001 and ****P < 0.0001.

We analyzed the expression levels of HRK in pan-cancerous tissues in the TCGA database through the online bioinformatics analysis website TIMER. We found that the expression level of HRK is heterogeneous in pan-cancer (**Figure 1A**). To explore the expression of HRK in CRC, we used Real-time RT-PCR and Western blotting in 8 CRC cell lines, an immortalized colon mucosa epithelial cell line (FHC) and paired CRC and adjacent noncancerous mucosa tissues.Compared with FHC, HRK mRNA expression was down regulated in CRC cell lines (**Figure 2A**). Western blotting also showed a decrease in HRK protein levels in CRC cell lines (**Figure 2B**). Consistent with the cell line results, reduced HRK mRNA (**Figure 2C**) and protein expression (**Figure 2D**) were observed in CRC tissues compared to paired non-cancerous mucosal tissues.

For further validation, we found that HRK mRNA was also downregulated in published CRC datasets (GEPIA, GSE87211) (**Figures 1B, C**). The immunohistochemistry (IHC) found that HRK expression was lower in colorectal cancer tissues compared to normal intestinal epithelium (**Figure 2E**). HRK was highly expressed in 73.17% (30 of 41) of the normal tissues and lowly expressed in 26.83% (11of 41). In cancer tissues, HRK was highly expressed in 32.26% (10 of 31) of cases and lowly expressed in 67.74% (21 of 31) (*P <*0.05, **Figure 2F**). By analyzing the public datasets GSE87211, it was revealed that the expression level of HRK was negatively correlated with distal metastasis. This suggests that colorectal cancer patients with lower HRK expression are more likely to occur with distant metastases. (**Figure 2G**, *P <*0.005). Overall survival from GSE17537 indicated that higher HRK expression was significantly correlated with longer survival in CRC patients (**Figure 2H**, log-rank test = 4.634, *P* =0.031).

### Knockdown of HRK enhances CRC cell proliferation, invasion and migration

**Figure 3:**
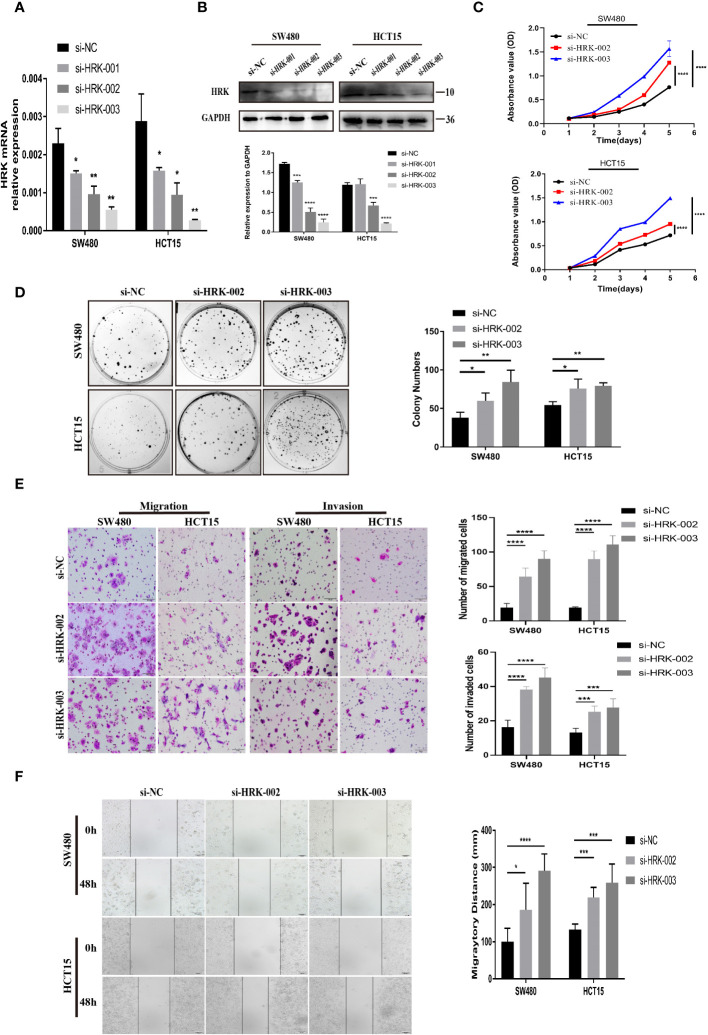
HRK reduction mediated by siRNA promotes CRC cell proliferation, migration, and invasion *in vitro*. **(A, B)**, knockdown expression of HRK after transfection with siRNA was confirmed in 2 CRC cell lines by Real time RT-PCR and western blotting. **(C)**, HRK reduction promoted cell proliferation of HCT15 and SW480 cells. **(D)**, Depletion of HRK increased the ability of HCT15 and SW480 cells to form colonies. **(E)**, Matrigel transwell assays were used to determine the invasion ability of HRK-downregulated CRC cells. Matrigel-free transwell assays showing the migration ability of HRK-downregulated CRC cells. **(F)**, Wound healing assays assessed the migration capability of HRK-downregulated CRC cells. Data are presented as the mean ± SD. The results were reproducible in 3 independent experiments. *P < 0.05, **P < 0.01, ***P <0.001 and **** *P <*0.0001.

The two siRNAs with the highest efficiency of HRK knockdown were screened by Real-time RT-PCR and Western blotting (**Figures 3A, B**). Then, two HRK knocked down cell lines were established with SW480 and HCT15 cells (**Figures 3A, B**), which had higher and consistent expression of endogenous HRK mRNA and protein levels (**Figures 2A, B**). Reduction of HRK led to an obvious impairment of proliferation and colony formation in CRC cells (**Figures 3C, D**). Next, we used Matrigel invasion assay, Matrigel-free transwell assay and Wound healing assay to explore the invasion and migration ability of HRK down-regulated CRC cells. The migration and invasion rate was significantly higher in the HRK low expression group compared to the control (**Figures 3E, F**). Collectively, it demonstrated that downregulation of HRK inhibited the proliferation, invasion and migration of CRC cells.

### Overexpression of HRK reduces CRC cell proliferation and migration *in vitro*

**Figure 4:**
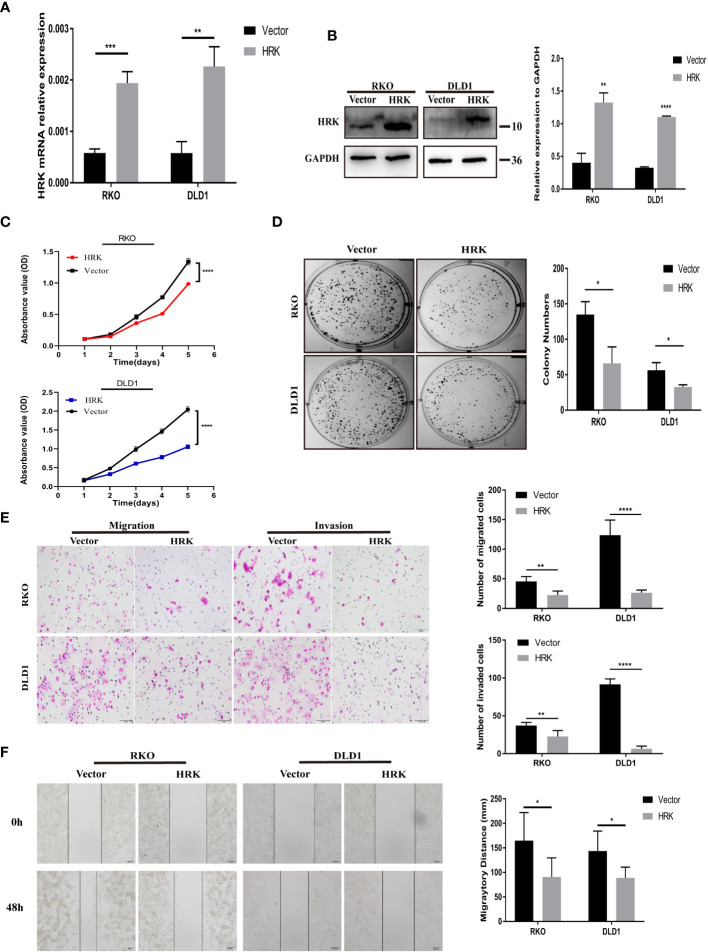
HRK overexpression reduces CRC cell proliferation, migration, and invasion *in vitro*. **(A, B)**, Overexpression of HRK after transfection of the pcDNA3.1-HRK or pcDNA3.1 plasmids was confirmed in 2 CRC cell lines by Real time RT-PCR and Western blotting. **(C, D)**, Effects of HRK overexpression on CRC cell proliferation by CCK-8 **(C)** and colony formation assays **(D, E)**, Matrigel transwell assays were used to determine the invasion ability of HRK-overexpressed CRC cells. Matrigel-free transwell assays showing the migration ability of HRK-overexpressed CRC cells. **(F)**, The effects of HRK overexpression on the migration abilities of CRC cells by using wound-healing assay. Data are expressed as the means ± SD in 3 independent experiments. *P <0.05, **P <0.01, ***P < 0.001 and ****P <0.0001.

The endogenous mRNA and protein expression levels of HRK in RKO and DLD1 cells were consistent and relatively low (**Figures 2A, B**). Therefore, these two cells were selected to transfect pcDNA 3.1-HRK or pcDNA 3.1 plasmid to establish functional acquisition model to verify the effect of HRK overexpression on the biological function of CRC cells *in vitro*. Real-time RT-PCR and Western blotting analysis proved that HRK was significantly upregulated in the cells compared to controls (**Figures 4A, B**). As shown by CCK-8 and colony formation, comparing with the control group, the growth rate of CRC cells was evidently inhibited after overexpressed HRK (**Figures 4C, D**) In addition, we also tested the ability of cell migration and invasion by Matrigel invasion assay, Matrigel-free transwell assay and Wound-healing assay. The invasiveness and motility of the cells were significantly reduced by HRK overexpression compared to the control (**Figures 4E, F**). The results mentioned above confirmed that upregulation of HRK reduces cell proliferation, migration and invasion *in vitro*, and may be involved in the physiological control of these processes, repressing carcinogenesis and tumor progression.

### HRK regulates CRC Cell cycle and Apoptosis

**Figure 5:**
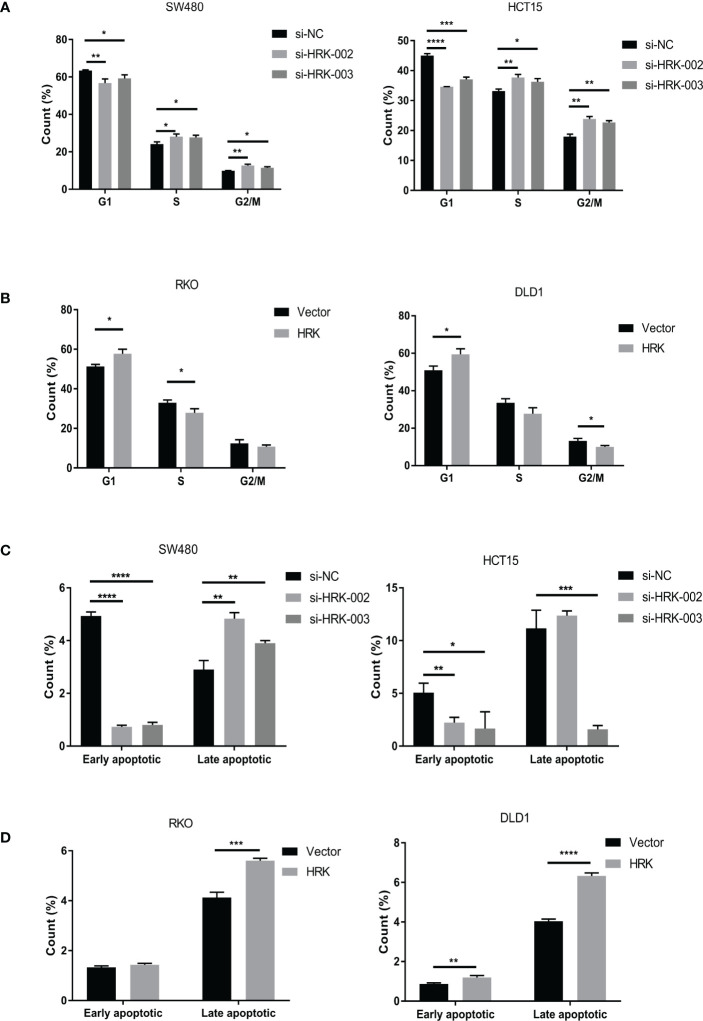
HRK regulates CRC Cell cycle and Apoptosis.**(A)**, The effects of HRK reduction on cell cycle progression by flow cytometry. **(B)**, The effects of HRK overexpression on cell cycle progression by flow cytometry. **(C)**, The effects of HRK reduction on cell apoptosis by flow cytometry. **(D)**, The effects of HRK overexpression on cell apoptosis by flow cytometry. Data are expressed as the means ± SD in 3 independent experiments. *P <0.05, **P <0.01, ***P < 0.001 and ****P <0.0001.

Cell cycle and apoptosis were detected according to flow cytometry. After reduced the expression of HRK in HCT15 and SW480, it showed an increased percentage of S and G2/M phases (**Figures 5A**, **S1A**) and decreased induction of early apoptosis (**Figures 5C**, **S1C**). Reduction of late apoptosis was seen only in HCT15 cells silenced with si-HRK- 003 (**Figures 5C**, **S1C**). Contrarily, cells transfected with pcDNA3.1-HRK plasmid induced G1 phase cell-cycle arrest in RKO as well as DLD1 cells (**Figures 5B**, **S1B**). HRK raised the percentage of late apoptosis in RKO as well as DLD1 cell lines (**Figures 5D**, S1D). Meanwhile, the percentage of early apoptosis in DLD1 was more than control (**Figures 5D**, **S1D**).

**Figure 6:**
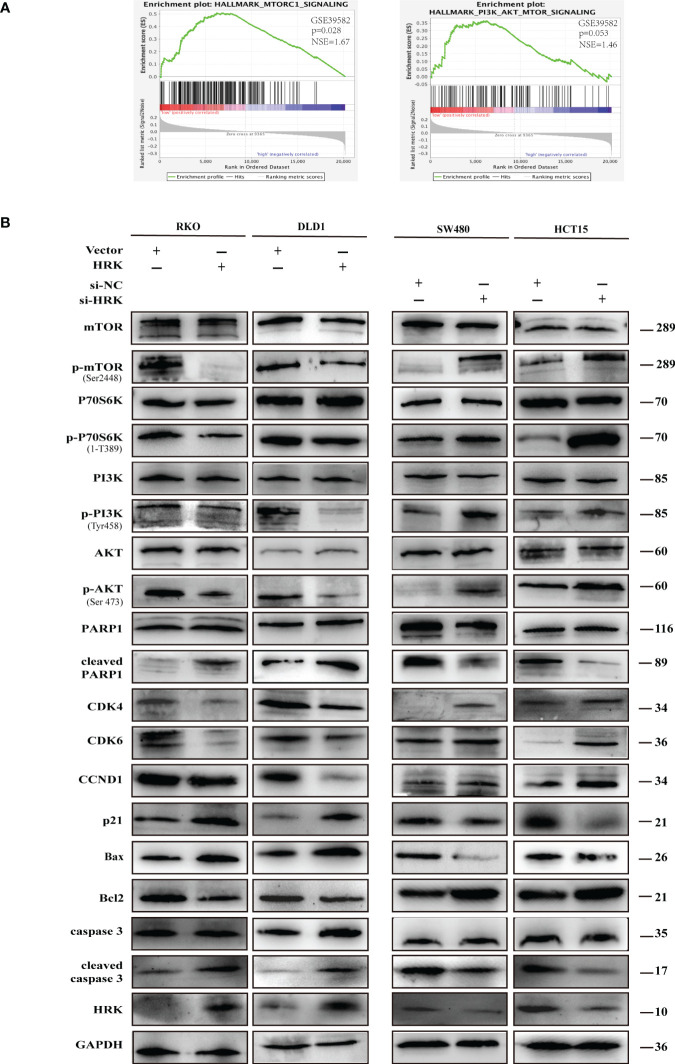
HRK regulates CRC processes through PI3K/AKT/mTOR signaling pathway. **(A)**, GSEA showed the enrichment of mTORC1 and PI3K/AKT/mTOR pathway in CRC cells with HRK downregulation. **(B)**, Western blotting was performed to detect the leves of mTOR, p-mTOR, PI3K, p-PI3K, P70S6K, p-P70S6K, AKT, p-AKT, PARP1, cleaved PARP1, CDK4, CDK6, Cylin D1, P21, Bax, Bcl-2, caspase3 and cleaved caspase3 in CRC cells that overexpress or downregulate HRK. + represents the use of the material. - means that the material is not used.

Western blotting proved that the proteins promoting cell cycle progression CDK4, CDK6 and Cyclin D1 were increased in the HRK downregulated cells SW480 and HCT15(**Figures 6B**, **S2A**). Nevertheless, p21, which inhibits cell cycle progression, was decreased after reduced the expression of HRK (**Figures 6B**, **S2A**). In addition, the anti-apoptotic protein Bcl2 was increased in both HRK downregulated cells SW480 and HCT15, whereas apoptosis-promoting proteins Bax, cleaved caspase-3 and cleaved PARP1 were all decreased (**Figures 6B**, **S2A**). However, when HRK expression was overexpressed in DLD1 as well as RKO cell lines we obtained the opposite results (**Figures 6B**, **S2A**). These data proved that HRK could modulate the apoptosis as well as cell cycle in CRC cells.

### HRK regulates CRC processes through PI3K/AKT/mTOR signaling pathway

**Figure 7:**
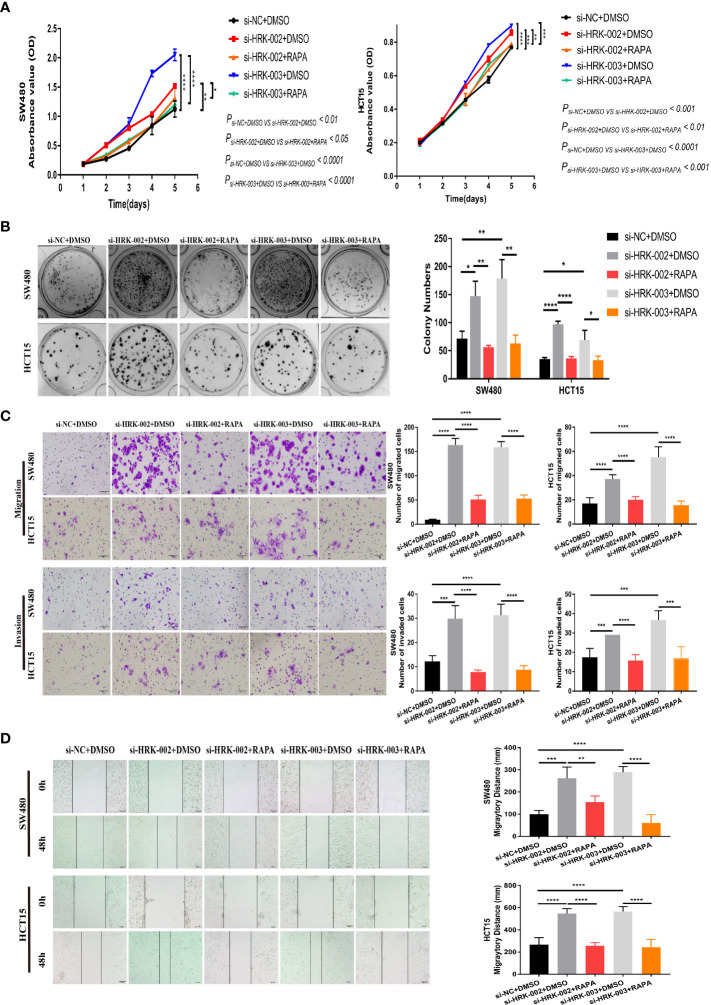
*In vitro* proliferation, invasion and migration ability of HRK expression-reduced cell lines were inhibited by rapamycin. A, B, CCK-8 **(A)** and colony formation assays **(B)** were used to evaluate the proliferation of CRC cells after HRK reduction or RAPA (100 nM) treatment for 24 h. **(C)**, Matrigel transwell assays and Matrigel-free transwell assays were used to evaluate the invasion and migration ability of CRC cells after HRK knockdown or RAPA (100 nM) treatment for 24 h. **(D)**, Wound-healing assay were used to evaluate the migration ability of CRC cells after HRK knockdown or RAPA (100 nM) treatment for 24 h. Data are expressed as the means ± SD in 3 independent experiments. *P <0.05, **P <0.01, ***P <0.001 and ****P < 0.0001.

**Figure 8:**
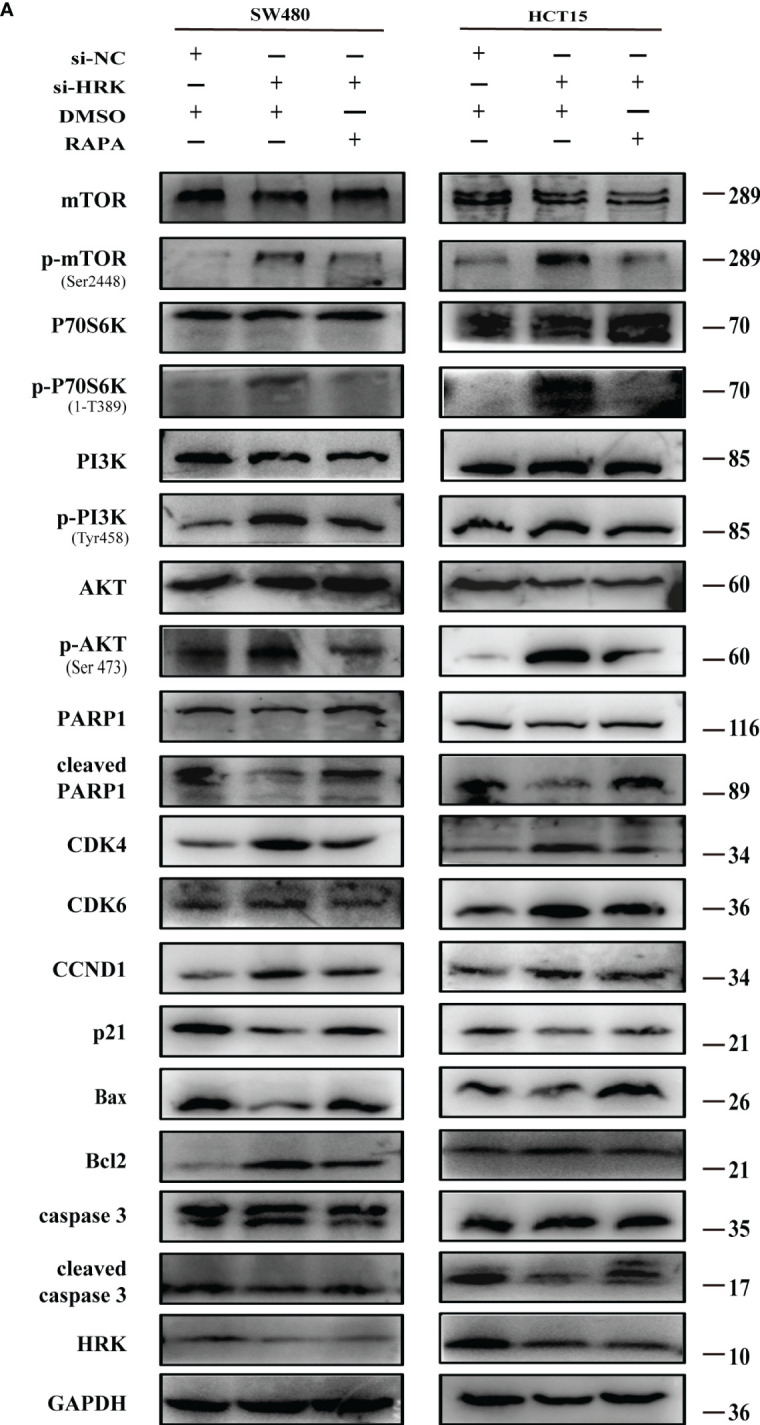
Effect of rapamycin on PI3K/AKT/mTOR signaling pathway in cell lines with reduced HRK expression **(A)**, Western blotting was performed to detect mTOR, p-mTOR, PI3K, p-PI3K, P70S6K, p-P70S6K, AKT, p-AKT, PARP1, cleaved PARP1, CDK4, CDK6, CCND1, P21, Bax, Bcl-2, caspase3 and cleaved caspase3 in CRC cells after HRK knockdown or RAPA (100 nM) treatment for 24 h. + represents the use of the material. - means that the material is not used.

To seek the signaling involved in HRK, we applied GSEA to analyze HRK from a data with 585 patients GSE39582, which was downloaded from Pubmed’s GEO database. According to the analysis, MTORC1 and PI3K/AKT/mTOR signaling pathways were enriched in the HRK low expression group (**Figure 6A**; NES=1.67; p=0.028; NES=1.46; p=0.053). As shown in **Figure 6B**, we analyzed the phosphorylation and subsequent activation of PI3K/AKTT/mTOR signaling pathway-related proteins. Downregulation of HRK in SW480 and HCT15 cells showed increased expression of phosphorylated PI3K, phosphorylated AKT, phosphorylated mTOR and phosphorylated P70S6K in the PI3K/AKT/mTOR signaling pathway (**Figures 6B**, **S2A**). Even more, we got the opposite result after upregulate the expression of HRK in DLD1 and RKO cells (**Figures 6B**, **S2A**). This indicates that HRK is involved in regulating the PI3K/AKT/mTOR signaling pathway. Rapamycin was used in CRC cells after HRK downregulated for 24 h. The promotion of CRC cell proliferation, migration and invasion caused by HRK knockdown was reversed by RAPA treatment (**Figures 7A–D**). Furthermore, western blotting suggested that rapamycin application reversed P70S6K, mTOR, PI3K and AKT phosphorylation and reduced the expression of cell cycle proteins CDK4, CDK6 and Cyclin D1 (**Figures 8A**, **S2B**). It increased the expression of Bax, cleaved caspase3, cleaved PARP1 and p21. In addition, it also decreased the expression of Bcl-2. Hence, rapamycin restored the ability of CRC cells apoptosis and proliferation (**Figures 8A**, **S2B**). In brief, the data suggest that HRK suppress CRC cells proliferation, migration and invasion by suppressing PI3K/AKT/mTOR signaling pathway.

## Discussion

HRK encodes a member of the BCL-2 protein family, which is mainly involved in activation or inhibition of apoptosis. Loss of HRK expression has been reported in a variety of carcinomas (14). It has been reported that HRK is deficient in expression in colorectal and gastric cancers (15). According to GSE87211 and GSE39582, we found that the mRNA expression of HRK in colorectal cancer was lower than adjacent normal mucosa. We also found that patients with higher expression of HRK had a better survival rate according to GSE17537. Therefore, we used fresh CRC tissues and matched normal mucosa to detect the expression level of HRK at the mRNA and protein levels. The expression of HRK was lower in colorectal cancer than that in normal tissues. These results were also confirmed by immunohistochemistry. Combined with the previous results, HRK may be a marker to indicate the prognosis of colorectal cancer. To elucidate the biological function of HRK in colorectal cancer, we upregulated HRK expression in colorectal cancer cells and found that increased HRK expression slowed down the proliferation of colorectal cancer cells and also reduced the invasion and migration ability of colorectal cancer cells, while knocking down HRK expression in colorectal cancer cells showed the opposite result. The data suggest that in colorectal cancer cells, HRK acts as a tumor suppressor gene.

The Bcl-2 family is one of the most important protein families regulating apoptosis, and the homologous structure of its effect on apoptosis includes three structural domains. Among them, the conserved structural domain of BH-4 has an inhibitory effect on apoptosis, while the conserved structural domain of BH-3 plays a key role in promoting apoptosis (23–27). HRK contains a conserved structural domain of BH-3, which means that HRK could theoretically promote the apoptotic process of cells. Some studies have proved that high apoptotic index in prostate cancer is positively correlated with HRK expression (17, 28), and it has also been suggested in the literature that HRK plays a key role in the induction of JNK/mitochondria-dependent apoptosis in prostate cancer cells by 2-ME (17).

In our study, we found that high expression of HRK blocked CRC cells in the G1 phase and reduced the expression of CDK4, CDK6 and Cyclin D1. Not only that, it also increased the expression of Bax, cleaved caspase 3, cleaved PARP1 and p21 as well as decreased the expression of Bcl-2. Proteins containing only the BH3 domain can trigger the activation of the multi-BH structural domain protein Bax either directly *via* binding or *via* inhibition of anti-apoptotic BCL-2 family proteins. Once activated, it can induce the formation of apoptotic bodies through pro-cytochrome c release into the cytoplasm. In the apoptotic vesicle caspase 9 is activated, with further activation of caspase 3 and 7, leading to apoptosis (29–31). During apoptosis, caspase 3 cleaves PARP1, and the increase of cleaved PARP1 hinders DNA damage repair and further promotes apoptosis (32). Normally, p21 binds to CDKs to block CDK protein activity causing cell cycle arrest (33). However, increased AKT activity causes dysregulation of FOXO3 expression, which in turn inhibits p21 expression (33–35). Thus, our study demonstrated that HRK can induce apoptosis and block cell cycle progression.

Analysis of public dataset GSE39582 using GSEA revealed that low expression of HRK may enrich mTORC1 as well as PI3K/AKT signaling pathway. Both pathways are intracellular signaling pathways that respond to extracellular signals and promote metabolism, proliferation, cell survival, growth and angiogenesis. Activation of the pathways promotes phosphorylation of a series of downstream substrates (36–39). AKT is activated through phosphorylation, and one of the most conserved functions of AKT is to regulate the downstream mTOR signaling pathway. AKT regulates the mTOR pathway by phosphorylating TSC2, which blocks the negative regulation of small G protein Rheb by TSC1/2, thereby indirectly activating mTOR complex 1(mTORC1) to promote cell growth as well as regulate apoptosis (40–43). MTORC1 is a key regulator of translation initiation and ribosome biogenesis, and plays an evolutionarily conserved role in cell growth control. It activates P70S6K and eukaryotic initiation factor 4E (eIF4E)-binding protein 1 (4E-BP1) (38). P70S6K1 also enhances protein synthesis by activating eIF4B and regressing eIF4A inhibitor programmed cell death 4 (PDCD4) (43–45). Degradation of PDCD4 inhibits apoptosis (44). Moreover, the activated P70S6K can inhibit the function of mTORC2, thus cutting off PI3K signaling through a negative feedback loop (45). In CRC cells with low HRK expression, PI3K/AKT/mTOR activation was increased to promote cell proliferation and survival through intracellular signaling.

Apoptosis is a cellular death program that evolved by an organism during development or following cellular stress to eliminate unwanted or abnormal cells from the body. Cysteaspartase protease activity is critical for apoptosis. Once activated, cysteaspartase cleaves into hundreds of different proteins, leading to rapid cell death (30, 31). Furthermore, overexpression of anti-apoptotic Bcl-2 family proteins can largely enhancement tumor development induced by oncogenes, although excessive expression of anti-apoptotic Bcl-2 family proteins alone may not lead to tumor development comparable to that of oncogenes themselves (31). Both growth factor secretion including FOXOs (46, 47) and E2F1 (48) and the presence of other apoptotic stimuli can induce transcription of proteins containing only BH3 structures. In contrast, the activity of some transcription factors can be inhibited by AKT (47). Thus, increased AKT activation may reduce HRK transcription. We still need more experiments to clarify the effect of Akt activation on HRK expression.

In conclusion, our study found that HRK expression was reduced in colorectal cancer. Highlighting that low-expressed HRK prevents apoptosis as well as promotes the proliferation, invasion and migration of colorectal cancer cells through PI3K/AKT/mTOR signaling pathway. It is suggested that HRK downregulation is associated with development and progression of colorectal cancer, making this protein a promising target for blocking or reversing progression of colorectal cancer.

However, our study has limitations: first, the number of specimens from our CRC patients was relatively small. Second, we only studied silencing and overexpression of HRK *in vitro*. *In vivo* experiments need to be continued in future work.

## Data availability statement

The original contributions presented in the study are included in the article/**Supplementary materials**. Further inquiries can be directed to the corresponding author.

## Ethics statement

The studies involving human participants were reviewed and approved by The Ethics Committee of Southern Medical University. The patients/participants provided their written informed consent to participate in this study.

## Author contributions

HW designed the project. HW, YC, and QY carried out the experiments, wrote the manuscript as well as revised the manuscript. LC and PD collected the samples and interpreted part of data. All authors contributed to the article and approved the submitted version.
