# Supplementary figures and images for: HRK inhibits colorectal cancer cells proliferation by suppressing the PI3K/AKT/mTOR pathway
Source: Front Oncol. 2022 Dec 7;12:1053510. doi: 10.3389/fonc.2022.1053510 (PMC9769574; doi:10.3389/fonc.2022.1053510)

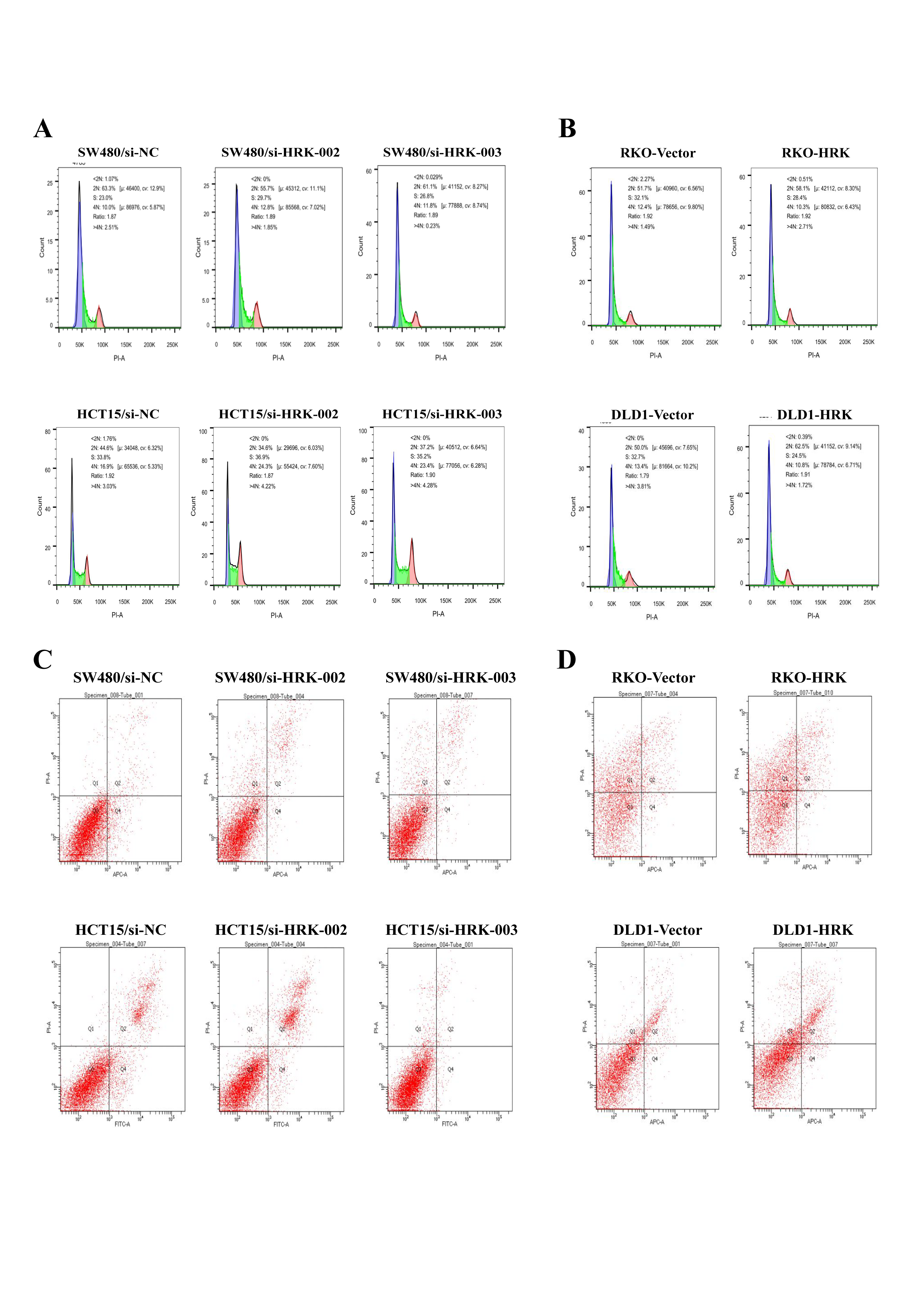

Supplement: Supplementary file 1 [file Image_1.tif]

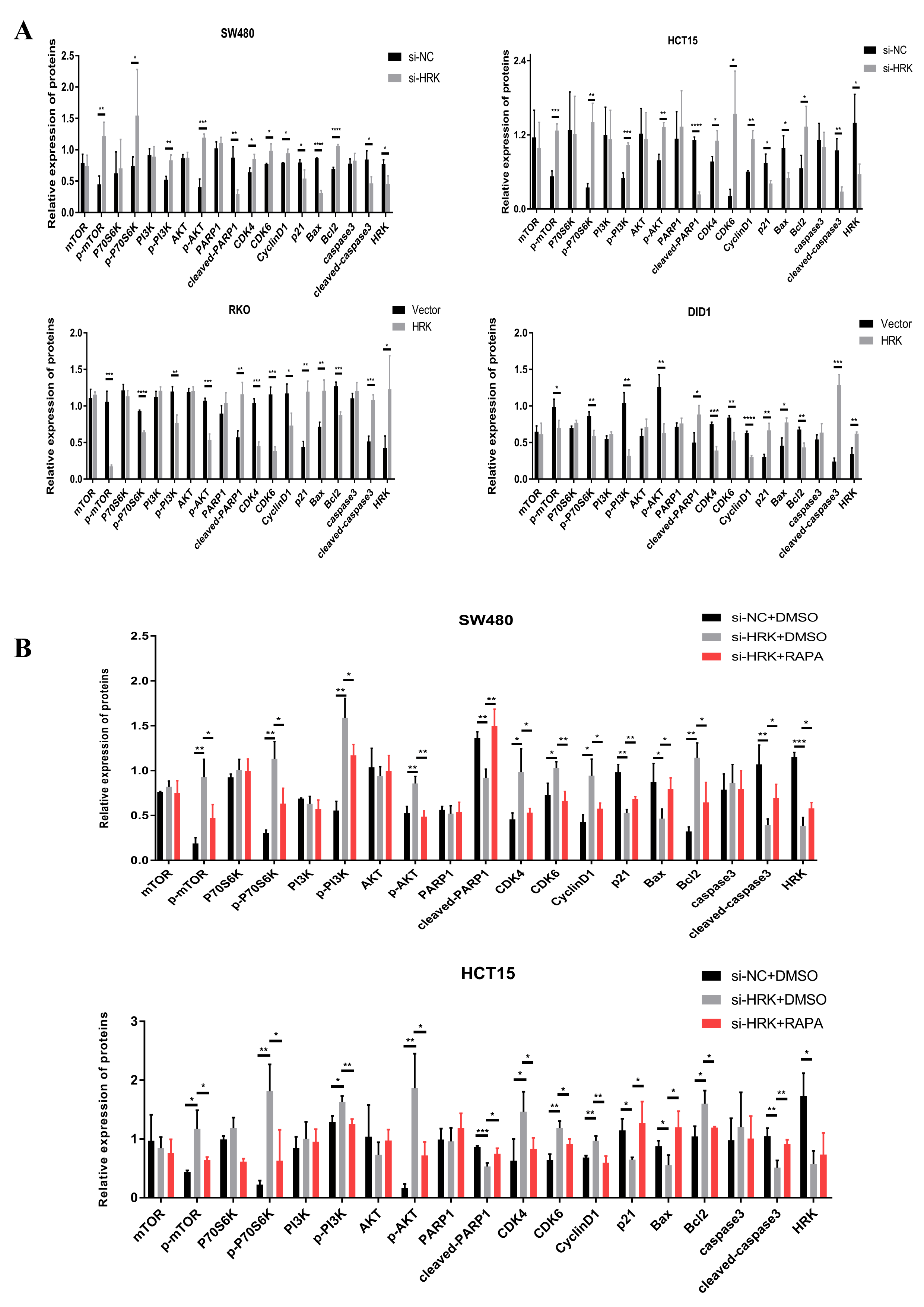

Supplement: Supplementary file 2 [file Image_2.tif]
