# Supplementary material for: HRK inhibits colorectal cancer cells proliferation by suppressing the PI3K/AKT/mTOR pathway
Source: Front Oncol. 2022 Dec 7;12:1053510. doi: 10.3389/fonc.2022.1053510 (PMC9769574; doi:10.3389/fonc.2022.1053510)
Supplement: Supplementary file 3 [file Table_1.docx]

**Data S1: Quantitative real-time PCR Primers:**

HRK:

Forward primer 5’-TGCTCGGCAGGCGGAACTTGTAG-3’

Reverse primer 5’-GCTTCCCCAGTCCCATTCTGTGTTT-3’

GAPDH:

Forward primer 5’-ACAACTTTGGTATCGTGGAAGG-3’

Reverse primer 5’-GCCATCACGCCACAGTTTC-3’

**Data S2: siRNA sequence of targeting HRK：**

si 1: 5’-TGCTCGGCAGGCGGAACTT-3’

si 2: 5’-CTGCACCAGCGCACCATGT-3’

si 3: 5’-GAGCGATCGTAGAAACACA-3’

Negative control sequence: belongs to RIBOBIO

**Data S3: Supplementary material and methods:**

**Cell proliferation and colony formation assays**

1×10^3^ cells per well were seeded and cultured into 96-well plates. Cell proliferation was assessed daily with the Cell Counting Kit (CCK-8) (NCM Biotech, USA) as described in the reagent instructions, and plates were read on a microplate reader at a wavelength of 450 nm.

Colony formation assay were performed in 6-well plates or 12-well plates with cells seeded at 5 × 10^2^ cells per well, cultured in RPMI 1640 containing 10% FBS. After 3 weeks of growth, cell colonies were fixed with methanol for 30 min and stained with Giemsa for 15 min. The surviving colonies (≥50 cells per colony) were then counted under a microscope. Experiments were carried out at least in triplicate.

**Wound-healing and transwell assays**

Cell migration was assessed by measuring cell movement toward the scraped cell-free area in a 6-well plate with a 10μL pipette. Culture with serum-free medium and observe the spread of wound closure every 24 hours. Migration was measured by calculating the distance of cells migrating toward the original wound area.

Cell invasion assay was performed through a 24-well plate containing 8 μm pores in a transwell chamber (BD Biosciences, San Jose, CA, USA). 1 × 10^5^ cells were starved overnight and seeded in 200 μL of serum-free medium into the upper chamber coated with Matrigel. The lower chamber was added with 500 μL of medium with 10% FBS. After incubated at 37 °C for 24 to 72 h, the non-invasive cells in the upper chamber were scraped off with a cotton swab, and the invasive cells adhering to the submembrane surface were fixed with methanol for 30 min and stained with crystal violet for 10 min. Cells were randomly counted in five regions of each membrane under a light microscope. Experiments were performed at least in triplicate.

Cell migration was performed through a 24-well plate containing 8 μm pores in a transwell chamber (BD Biosciences, San Jose, CA, USA). 1 × 10^5^ cells were seeded on the upper side of the membrane. The lower chamber was used with 10% FBS. Following 24-72 h at 37 °C. Cells adhering to the lower surface of the membrane were fixed with methanol for 30 min and stained with crystal violet for 10 min. Five areas were randomly selected for cell counting. The experiment was repeated at least 3 times.

**Cell apoptosis analysis**

For cell cycle analysis, cells were collected and fixed overnight at 4 °C in ice-cold 70% ethanol. Afterwards, cells were resuspended in propidium iodide solution (Genechem, Shanghai, China) and subjected to FACS analysis according to the manufacturing reagent instructions.

For apoptosis analysis, cells were digested with EDTA-free trypsin and collected. Subsequently, they were resuspended in binding buffer and stained with Annexin V-FITC assay kit (Genechem) according to the manufacturer's instructions. Apoptosis was analyzed by flow cytometry. Experiments were performed at least in triplicate.
